# Poly[octa-μ-aqua-tetra­aqua­bis(μ-5-sul­fon­atobenzene-1,3-dicarboxyl­ato)cobalt(II)tetra­sodium]

**DOI:** 10.1107/S1600536809008174

**Published:** 2009-03-11

**Authors:** Bing-Yu Zhang, Jing-Jing Nie, Duan-Jun Xu

**Affiliations:** a Department of Chemistry, Zhejiang University, People’s Republic of China

## Abstract

The title compound, [CoNa_4_(C_8_H_3_O_7_S)_2_(H_2_O)_12_]_*n*_, is a three-dimensional coordination polymer bridged by sulfoisophthalate trianions and water mol­ecules. The Co^II^ atom, located on an inversion centre, is coordinated by two carboxyl­ate groups of the sulfoisophthalate trianions and by four water mol­ecules in a distorted CoO_6_ octa­hedral geometry. Two independent Na^I^ atoms also have a distorted octa­hedral coordination geometry formed by water, carboxyl­ate O and sulfonate O atoms. An extensive O—H⋯O and C—H⋯O hydrogen-bonding network is present in the crystal structure, as well as weak π-π stacking [centroid–centroid distance = 3.9553 (11) Å].

## Related literature

For the role played by π–π stacking between aromatic rings in the electron-transfer process in some biological systems, see: Deisenhofer & Michel (1989 ▶); Su & Xu (2004 ▶); Liu *et al.* (2004 ▶); Pan *et al.* (2006 ▶). For a related structure, see: Zhang *et al.* (2008 ▶).
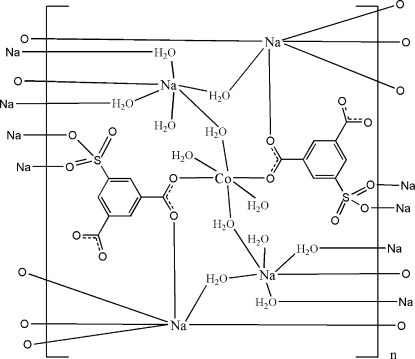


## Experimental

### 

#### Crystal data

[CoNa_4_(C_8_H_3_O_7_S)_2_(H_2_O)_12_]
*M* *_r_* = 853.41
Monoclinic, 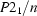

*a* = 7.8756 (12) Å
*b* = 17.294 (3) Å
*c* = 11.7700 (18) Å
β = 93.281 (5)°
*V* = 1600.5 (4) Å^3^
*Z* = 2
Mo *K*α radiation
μ = 0.82 mm^−1^
*T* = 295 K
0.35 × 0.32 × 0.25 mm

#### Data collection

Rigaku R-AXIS RAPID IP diffractometer
Absorption correction: multi-scan (**ABSCOR**; Higashi, 1995 ▶) *T*_min_ = 0.756, *T*_max_ = 0.819
9383 measured reflections
3120 independent reflections
2899 reflections with *I* > 2σ(*I*)
*R*_int_ = 0.016

#### Refinement

*R*[*F* ^2^ > 2σ(*F* ^2^)] = 0.025
*wR*(*F* ^2^) = 0.066
*S* = 1.08
3120 reflections
223 parameters
H-atom parameters constrained
Δρ_max_ = 0.42 e Å^−3^
Δρ_min_ = −0.40 e Å^−3^

### 

Data collection: *PROCESS-AUTO* (Rigaku, 1998 ▶); cell refinement: *PROCESS-AUTO*; data reduction: *CrystalStructure* (Rigaku/MSC, 2002 ▶); program(s) used to solve structure: *SHELXS97* (Sheldrick, 2008 ▶); program(s) used to refine structure: *SHELXL97* (Sheldrick, 2008 ▶); molecular graphics: *ORTEP-3 for Windows* (Farrugia, 1997 ▶); software used to prepare material for publication: *WinGX* (Farrugia, 1999 ▶).

## Supplementary Material

Crystal structure: contains datablocks I, global. DOI: 10.1107/S1600536809008174/is2394sup1.cif

Structure factors: contains datablocks I. DOI: 10.1107/S1600536809008174/is2394Isup2.hkl

Additional supplementary materials:  crystallographic information; 3D view; checkCIF report

## Figures and Tables

**Table 1:** Selected bond lengths (Å)

Co—O1	2.0541 (11)
Co—O8	2.1039 (13)
Co—O9	2.1122 (11)
Na1—O5^i^	2.3477 (13)
Na1—O9	2.4322 (13)
Na1—O10	2.4702 (14)
Na1—O11	2.5343 (15)
Na1—O12	2.4048 (14)
Na1—O13	2.4037 (15)
Na2—O2	2.4557 (13)
Na2—O5^ii^	2.4785 (13)
Na2—O6^iii^	2.4344 (13)
Na2—O10	2.4787 (15)
Na2—O11^iv^	2.3506 (14)
Na2—O12^iv^	2.5222 (14)

Symmetry codes: (i) 
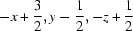
; (ii) 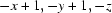; (iii) 
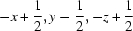
; (iv) 
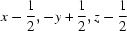
.

**Table 2:** Hydrogen-bond geometry (Å, °)

*D*—H⋯*A*	*D*—H	H⋯*A*	*D*⋯*A*	*D*—H⋯*A*
O8—H8*A*⋯O13^v^	0.84	2.02	2.8584 (19)	172
O8—H8*B*⋯O4^ii^	0.85	1.98	2.8028 (18)	160
O9—H9*A*⋯O7^iii^	0.86	2.16	2.9932 (16)	164
O9—H9*B*⋯O2	0.84	1.83	2.6222 (16)	158
O10—H10*A*⋯O7^ii^	0.83	2.04	2.8602 (17)	168
O10—H10*B*⋯O3^vi^	0.85	1.84	2.6676 (18)	165
O11—H11*A*⋯O7^iii^	0.89	1.89	2.7621 (17)	167
O11—H11*B*⋯O3^vi^	0.87	1.92	2.7904 (19)	175
O12—H12*A*⋯O1^vii^	0.84	2.12	2.9531 (17)	174
O12—H12*B*⋯O4^viii^	0.89	2.05	2.9076 (18)	162
O13—H13*A*⋯O4^ii^	0.84	1.93	2.7277 (18)	157
O13—H13*B*⋯O6^i^	0.88	2.22	2.9597 (17)	142
C7—H7⋯O11^iv^	0.93	2.49	3.371 (2)	157

Symmetry codes: (i) 
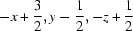
; (ii) 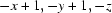; (iii) 
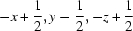
; (iv) 
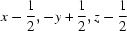
; (v) 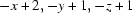; (vi) 
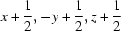
; (vii) 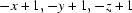; (viii) 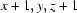.

**Fig. 1.**
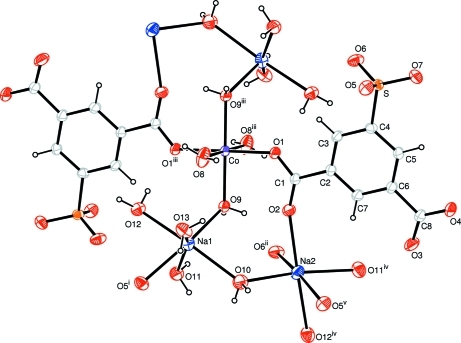
A part of the polymeric structure of the title compound with 50% probability displacement (arbitrary spheres for H atoms) [symmetry codes: (i) -x + 3/2,+y - 1/2,-z + 1/2; (ii) -x + 1/2,+y - 1/2,-z + 1/2; (iii) -x + 1,-y + 1,-z + 1; (iv) x - 1/2,-y + 1/2,+z - 1/2; (v) -x + 1,-y + 1,-z].

**Fig. 2.**
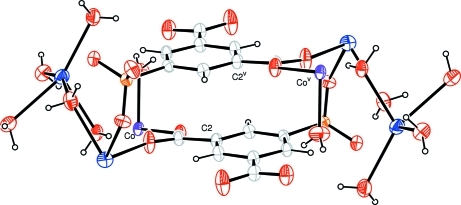
π-π stacking between benzene rings [symmetry code: (v) -x + 1,-y + 1,-z].

## supplementary crystallographic information

### Comment

As π-π stacking between aromatic rings plays an important role in electron transfer process in some biological system (Deisenhofer & Michel, 1989), π-π stacking has attracted our much attention in past years (Su & Xu, 2004; Liu *et al.*, 2004; Pan *et al.*, 2006). In order to investigate the influence of substituents of the aromatic compounds on stacking between parallel aromatic rings, the title Co^II^ compound incorporating sulfoisophthalate ligand has recently been prepared and its crystal structure is reported here.

The title compound is a three dimensional polymeric complex bridged by sulfoisophthalate trianions and water molecules (Fig. 1). The Co atom occupies a special position in an inversion centre and is coordinated by four water molecules and two carboxyl groups from sulfoisophthalate trianions in a distorted CoO_6_ octahedral geometry (Table 1). The crystal structure contains two independent Na^I^ atoms, both in distorted octahedral coordination geometry. The Na1 atom is coordinated by five water molecules and one sulfonate O atom, while the Na2 atom is coordinated by three water molecules, two sulfonate O atoms and one carboxy O atom. The C8-carboxy group of the sulfoisophthalate ligand is not coordinated to the metal atom in the structure.

The extensive O—H···O hydrogen bonding network presents in the crystal structure (Table 2), weak C—H···O hydrogen bonding also helps to stabilize the crystal structure. A partial overlapped arrangement between centro-symmetric benzene rings is observed in the crystal structure (Fig. 2), perpendicular distance of the centroid of the C2-benzne ring on the C2^v^benzene ring is 3.563 Å, [symmetry code: (v) 1 - *x*,1 - *y*,-*z*], which suggests a weak π-π stacking involving sulfoisophthalate ligand (Zhang *et al.*, 2008).

### Experimental

A water-ethanol solution (25 ml, 3:2) containing monosodium 5-sulfoisophthalate (0.270 g, 1 mmol), Na_2_CO_3_ (0.212 g, 2 mmol), NaOH (0.081 g, 2 mmol) and CoCl_2_.6H_2_O (0.595 g, 2.5 mmol) was refluxed for 7.5 h and filtered after cooling to room temperature. The single crystals of the title compound were obtained from the filtrate after one month.

### Refinement

Water H atoms were located in a difference Fourier map and refined as riding in as-found relative positions, with *U*_iso_(H) = 1.5*U*_eq_(O). Other H atoms were placed in calculated positions (C—H = 0.93 Å) and refined in riding mode, with *U*_iso_(H) = 1.2*U*_eq_(C).

### Crystal data

**Table:**

[CoNa_4_(C_8_H_3_O_7_S)_2_(H_2_O)_12_]	*F*(000) = 874
*M**_r_* = 853.41	*D*_x_ = 1.771 Mg m^−^^3^
Monoclinic, *P*2_1_/*n*	Mo *K*α radiation, λ = 0.71073 Å
Hall symbol: -P 2yn	Cell parameters from 2508 reflections
*a* = 7.8756 (12) Å	θ = 2.5–25.0°
*b* = 17.294 (3) Å	µ = 0.82 mm^−^^1^
*c* = 11.7700 (18) Å	*T* = 295 K
β = 93.281 (5)°	Chuck, red
*V* = 1600.5 (4) Å^3^	0.35 × 0.32 × 0.25 mm
*Z* = 2	

### Data collection

**Table:**

Rigaku R-AXIS RAPID IP diffractometer	3120 independent reflections
Radiation source: fine-focus sealed tube	2899 reflections with *I* > 2σ(*I*)
graphite	*R*_int_ = 0.016
Detector resolution: 10.0 pixels mm^-1^	θ_max_ = 26.0°, θ_min_ = 2.1°
ω scans	*h* = −9→9
Absorption correction: multi-scan (*ABSCOR*; Higashi, 1995)	*k* = −21→21
*T*_min_ = 0.756, *T*_max_ = 0.819	*l* = −14→14
9383 measured reflections	

### Refinement

**Table:**

Refinement on *F*^2^	Primary atom site location: structure-invariant direct methods
Least-squares matrix: full	Secondary atom site location: difference Fourier map
*R*[*F*^2^ > 2σ(*F*^2^)] = 0.025	Hydrogen site location: inferred from neighbouring sites
*wR*(*F*^2^) = 0.066	H-atom parameters constrained
*S* = 1.08	*w* = 1/[σ^2^(*F*_o_^2^) + (0.035*P*)^2^ + 0.6342*P*] where *P* = (*F*_o_^2^ + 2*F*_c_^2^)/3
3120 reflections	(Δ/σ)_max_ = 0.002
223 parameters	Δρ_max_ = 0.42 e Å^−^^3^
0 restraints	Δρ_min_ = −0.40 e Å^−^^3^

### Special details

**Table:**

Geometry. All e.s.d.'s (except the e.s.d. in the dihedral angle between two l.s. planes) are estimated using the full covariance matrix. The cell e.s.d.'s are taken into account individually in the estimation of e.s.d.'s in distances, angles and torsion angles; correlations between e.s.d.'s in cell parameters are only used when they are defined by crystal symmetry. An approximate (isotropic) treatment of cell e.s.d.'s is used for estimating e.s.d.'s involving l.s. planes.
Refinement. Refinement of *F*^2^ against ALL reflections. The weighted *R*-factor *wR* and goodness of fit *S* are based on *F*^2^, conventional *R*-factors *R* are based on *F*, with *F* set to zero for negative *F*^2^. The threshold expression of *F*^2^ > σ(*F*^2^) is used only for calculating *R*-factors(gt) *etc*. and is not relevant to the choice of reflections for refinement. *R*-factors based on *F*^2^ are statistically about twice as large as those based on *F*, and *R*- factors based on ALL data will be even larger.

### Fractional atomic coordinates and isotropic or equivalent isotropic displacement parameters (Å^2^)

**Table:**

	*x*	*y*	*z*	*U*_iso_*/*U*_eq_	
Co	0.5000	0.5000	0.5000	0.01857 (9)	
Na1	0.82210 (8)	0.33026 (4)	0.45527 (6)	0.02963 (16)	
Na2	0.40642 (9)	0.26855 (4)	0.19976 (5)	0.02861 (16)	
S	0.28585 (5)	0.72991 (2)	0.01806 (3)	0.01880 (10)	
O1	0.42263 (15)	0.52219 (6)	0.33357 (9)	0.0254 (2)	
O2	0.42504 (18)	0.40595 (6)	0.25165 (9)	0.0338 (3)	
O3	0.1617 (2)	0.37979 (7)	−0.13019 (11)	0.0444 (4)	
O4	0.11425 (17)	0.47988 (7)	−0.24435 (10)	0.0324 (3)	
O5	0.45517 (15)	0.74752 (7)	−0.01734 (10)	0.0274 (3)	
O6	0.25309 (16)	0.76048 (6)	0.12948 (10)	0.0294 (3)	
O7	0.15486 (14)	0.75313 (6)	−0.06860 (10)	0.0266 (3)	
O8	0.75313 (16)	0.52064 (8)	0.46031 (11)	0.0384 (3)	
H8A	0.8214	0.5439	0.5062	0.058*	
H8B	0.7812	0.5310	0.3929	0.058*	
O9	0.53565 (14)	0.38179 (6)	0.46304 (9)	0.0240 (2)	
H9A	0.4672	0.3521	0.4963	0.036*	
H9B	0.5048	0.3772	0.3941	0.036*	
O10	0.70112 (17)	0.26175 (7)	0.28516 (10)	0.0334 (3)	
H10A	0.7540	0.2617	0.2263	0.050*	
H10B	0.6997	0.2140	0.3027	0.050*	
O11	0.68645 (15)	0.21915 (7)	0.55730 (11)	0.0322 (3)	
H11A	0.5780	0.2260	0.5709	0.048*	
H11B	0.6804	0.1860	0.5018	0.048*	
O12	0.86980 (15)	0.37242 (7)	0.64918 (10)	0.0315 (3)	
H12A	0.7916	0.4045	0.6573	0.047*	
H12B	0.9542	0.3962	0.6885	0.047*	
O13	1.03883 (16)	0.40306 (7)	0.36711 (11)	0.0354 (3)	
H13A	1.0031	0.4325	0.3141	0.053*	
H13B	1.1113	0.3713	0.3371	0.053*	
C1	0.4040 (2)	0.47732 (9)	0.24890 (13)	0.0208 (3)	
C2	0.3461 (2)	0.51502 (9)	0.13824 (13)	0.0210 (3)	
C3	0.3431 (2)	0.59494 (8)	0.12797 (12)	0.0208 (3)	
H3	0.3844	0.6260	0.1879	0.025*	
C4	0.27793 (19)	0.62793 (8)	0.02779 (12)	0.0190 (3)	
C5	0.2182 (2)	0.58293 (9)	−0.06349 (13)	0.0212 (3)	
H5	0.1747	0.6061	−0.1303	0.025*	
C6	0.2241 (2)	0.50268 (8)	−0.05391 (13)	0.0222 (3)	
C7	0.2891 (2)	0.46985 (9)	0.04690 (13)	0.0238 (3)	
H7	0.2944	0.4163	0.0532	0.029*	
C8	0.1620 (2)	0.45023 (9)	−0.15059 (13)	0.0244 (3)	

### Atomic displacement parameters (Å^2^)

**Table:**

	*U*^11^	*U*^22^	*U*^33^	*U*^12^	*U*^13^	*U*^23^
Co	0.02424 (17)	0.01734 (15)	0.01366 (15)	0.00223 (11)	−0.00299 (11)	−0.00032 (10)
Na1	0.0267 (4)	0.0352 (4)	0.0267 (3)	0.0019 (3)	−0.0009 (3)	0.0029 (3)
Na2	0.0307 (4)	0.0317 (4)	0.0234 (3)	−0.0005 (3)	0.0014 (3)	−0.0039 (3)
S	0.0222 (2)	0.01579 (17)	0.01856 (19)	−0.00060 (13)	0.00249 (14)	0.00052 (13)
O1	0.0398 (7)	0.0203 (5)	0.0152 (5)	0.0030 (5)	−0.0061 (5)	−0.0009 (4)
O2	0.0610 (9)	0.0191 (6)	0.0200 (6)	0.0054 (5)	−0.0091 (5)	0.0002 (4)
O3	0.0828 (11)	0.0193 (6)	0.0289 (7)	−0.0026 (6)	−0.0164 (7)	−0.0035 (5)
O4	0.0490 (8)	0.0294 (6)	0.0178 (6)	0.0020 (5)	−0.0087 (5)	−0.0020 (5)
O5	0.0246 (6)	0.0290 (6)	0.0288 (6)	−0.0064 (5)	0.0045 (5)	−0.0014 (5)
O6	0.0445 (7)	0.0214 (5)	0.0233 (6)	−0.0008 (5)	0.0098 (5)	−0.0034 (4)
O7	0.0267 (6)	0.0243 (5)	0.0284 (6)	0.0028 (5)	−0.0010 (5)	0.0059 (5)
O8	0.0302 (7)	0.0608 (8)	0.0242 (6)	−0.0087 (6)	0.0021 (5)	−0.0028 (6)
O9	0.0324 (6)	0.0194 (5)	0.0196 (5)	0.0014 (4)	−0.0048 (4)	−0.0005 (4)
O10	0.0469 (8)	0.0290 (6)	0.0246 (6)	0.0013 (5)	0.0035 (5)	0.0039 (5)
O11	0.0273 (7)	0.0309 (6)	0.0379 (7)	0.0038 (5)	−0.0036 (5)	−0.0058 (5)
O12	0.0329 (7)	0.0296 (6)	0.0315 (6)	0.0027 (5)	−0.0013 (5)	−0.0076 (5)
O13	0.0377 (7)	0.0306 (6)	0.0380 (7)	0.0067 (5)	0.0035 (6)	0.0064 (5)
C1	0.0270 (8)	0.0190 (7)	0.0159 (7)	0.0000 (6)	−0.0023 (6)	0.0005 (6)
C2	0.0270 (8)	0.0202 (7)	0.0155 (7)	0.0008 (6)	−0.0020 (6)	−0.0001 (6)
C3	0.0268 (8)	0.0199 (7)	0.0155 (7)	−0.0013 (6)	−0.0002 (6)	−0.0027 (6)
C4	0.0219 (8)	0.0158 (7)	0.0196 (7)	−0.0004 (6)	0.0026 (6)	0.0004 (6)
C5	0.0264 (8)	0.0210 (7)	0.0158 (7)	0.0004 (6)	−0.0018 (6)	0.0022 (6)
C6	0.0284 (9)	0.0216 (8)	0.0163 (8)	−0.0006 (6)	−0.0015 (6)	−0.0010 (6)
C7	0.0344 (9)	0.0171 (7)	0.0195 (8)	−0.0004 (6)	−0.0025 (6)	0.0006 (6)
C8	0.0315 (9)	0.0229 (8)	0.0182 (8)	0.0004 (6)	−0.0030 (6)	−0.0023 (6)

### Geometric parameters (Å, °)

**Table:**

Co—O1^i^	2.0541 (11)	O4—C8	1.2550 (19)
Co—O1	2.0541 (11)	O8—H8A	0.8421
Co—O8	2.1039 (13)	O8—H8B	0.8549
Co—O8^i^	2.1039 (13)	O9—H9A	0.8546
Co—O9^i^	2.1122 (11)	O9—H9B	0.8373
Co—O9	2.1122 (11)	O10—H10A	0.8284
Na1—O5^ii^	2.3477 (13)	O10—H10B	0.8512
Na1—O9	2.4322 (13)	O11—H11A	0.8860
Na1—O10	2.4702 (14)	O11—H11B	0.8680
Na1—O11	2.5343 (15)	O12—H12A	0.8381
Na1—O12	2.4048 (14)	O12—H12B	0.8887
Na1—O13	2.4037 (15)	O13—H13A	0.8406
Na2—O2	2.4557 (13)	O13—H13B	0.8803
Na2—O5^iii^	2.4785 (13)	C1—C2	1.504 (2)
Na2—O6^iv^	2.4344 (13)	C2—C7	1.383 (2)
Na2—O10	2.4787 (15)	C2—C3	1.388 (2)
Na2—O11^v^	2.3506 (14)	C3—C4	1.382 (2)
Na2—O12^v^	2.5222 (14)	C3—H3	0.9300
S—O6	1.4508 (12)	C4—C5	1.387 (2)
S—O5	1.4519 (12)	C5—C6	1.393 (2)
S—O7	1.4647 (12)	C5—H5	0.9300
S—C4	1.7688 (14)	C6—C7	1.387 (2)
O1—C1	1.2648 (18)	C6—C8	1.514 (2)
O2—C1	1.2456 (19)	C7—H7	0.9300
O3—C8	1.242 (2)		
			
O1^i^—Co—O1	180.000 (1)	S—O6—Na2^vii^	153.39 (8)
O1—Co—O8	89.41 (5)	Co—O8—H8A	121.1
O1—Co—O8^i^	90.59 (5)	Co—O8—H8B	122.7
O8—Co—O8^i^	180.00 (8)	H8A—O8—H8B	107.8
O1—Co—O9^i^	88.85 (4)	Co—O9—Na1	119.79 (5)
O8—Co—O9^i^	91.17 (5)	Co—O9—H9A	113.1
O1—Co—O9	91.15 (4)	Na1—O9—H9A	114.1
O8—Co—O9	88.83 (5)	Co—O9—H9B	104.8
O9^i^—Co—O9	180.0	Na1—O9—H9B	98.5
O5^ii^—Na1—O13	85.25 (5)	H9A—O9—H9B	103.4
O5^ii^—Na1—O12	79.41 (5)	Na1—O10—Na2	127.93 (6)
O13—Na1—O12	100.06 (5)	Na1—O10—H10A	119.3
O5^ii^—Na1—O9	152.84 (5)	Na2—O10—H10A	99.5
O13—Na1—O9	120.49 (5)	Na1—O10—H10B	106.2
O12—Na1—O9	87.02 (4)	Na2—O10—H10B	96.8
O5^ii^—Na1—O10	101.92 (5)	H10A—O10—H10B	102.5
O13—Na1—O10	98.73 (5)	Na2^viii^—O11—Na1	87.47 (5)
O12—Na1—O10	161.21 (5)	Na2^viii^—O11—H11A	122.5
O9—Na1—O10	83.72 (4)	Na1—O11—H11A	114.9
O5^ii^—Na1—O11	73.63 (4)	Na2^viii^—O11—H11B	127.1
O13—Na1—O11	158.54 (5)	Na1—O11—H11B	98.6
O12—Na1—O11	80.06 (5)	H11A—O11—H11B	102.3
O9—Na1—O11	80.96 (4)	Na1—O12—Na2^viii^	86.59 (4)
O10—Na1—O11	82.35 (5)	Na1—O12—H12A	103.5
O11^v^—Na2—O6^iv^	101.54 (5)	Na2^viii^—O12—H12A	133.4
O11^v^—Na2—O2	96.94 (5)	Na1—O12—H12B	134.9
O6^iv^—Na2—O2	82.90 (4)	Na2^viii^—O12—H12B	104.6
O11^v^—Na2—O5^iii^	74.63 (5)	H12A—O12—H12B	99.6
O6^iv^—Na2—O5^iii^	169.14 (5)	Na1—O13—H13A	114.9
O2—Na2—O5^iii^	107.52 (5)	Na1—O13—H13B	109.8
O11^v^—Na2—O10	158.18 (5)	H13A—O13—H13B	106.1
O6^iv^—Na2—O10	100.25 (5)	O2—C1—O1	125.33 (14)
O2—Na2—O10	84.48 (5)	O2—C1—C2	119.03 (13)
O5^iii^—Na2—O10	84.15 (5)	O1—C1—C2	115.60 (13)
O11^v^—Na2—O12^v^	81.34 (4)	C7—C2—C3	119.46 (14)
O6^iv^—Na2—O12^v^	94.71 (4)	C7—C2—C1	119.83 (13)
O2—Na2—O12^v^	176.74 (5)	C3—C2—C1	120.67 (13)
O5^iii^—Na2—O12^v^	74.76 (4)	C4—C3—C2	119.29 (13)
O10—Na2—O12^v^	98.15 (4)	C4—C3—H3	120.4
O11^v^—Na2—Na1^v^	48.51 (4)	C2—C3—H3	120.4
O6^iv^—Na2—Na1^v^	126.05 (4)	C3—C4—C5	121.49 (13)
O2—Na2—Na1^v^	134.99 (4)	C3—C4—S	117.01 (11)
O5^iii^—Na2—Na1^v^	43.99 (3)	C5—C4—S	121.40 (11)
O10—Na2—Na1^v^	117.07 (4)	C4—C5—C6	119.21 (14)
O12^v^—Na2—Na1^v^	45.26 (3)	C4—C5—H5	120.4
O6—S—O5	113.37 (7)	C6—C5—H5	120.4
O6—S—O7	112.02 (7)	C7—C6—C5	119.09 (14)
O5—S—O7	111.38 (7)	C7—C6—C8	119.03 (13)
O6—S—C4	107.22 (7)	C5—C6—C8	121.88 (14)
O5—S—C4	105.31 (7)	C2—C7—C6	121.43 (14)
O7—S—C4	107.00 (7)	C2—C7—H7	119.3
C1—O1—Co	130.65 (10)	C6—C7—H7	119.3
C1—O2—Na2	161.18 (11)	O3—C8—O4	124.55 (15)
S—O5—Na1^vi^	136.11 (7)	O3—C8—C6	116.52 (14)
S—O5—Na2^iii^	132.98 (7)	O4—C8—C6	118.93 (14)
Na1^vi^—O5—Na2^iii^	88.86 (4)		

Symmetry codes: (i) −*x*+1, −*y*+1, −*z*+1; (ii) −*x*+3/2, *y*−1/2, −*z*+1/2; (iii) −*x*+1, −*y*+1, −*z*; (iv) −*x*+1/2, *y*−1/2, −*z*+1/2; (v) *x*−1/2, −*y*+1/2, *z*−1/2; (vi) −*x*+3/2, *y*+1/2, −*z*+1/2; (vii) −*x*+1/2, *y*+1/2, −*z*+1/2; (viii) *x*+1/2, −*y*+1/2, *z*+1/2.

### Hydrogen-bond geometry (Å, °)

**Table:**

*D*—H···*A*	*D*—H	H···*A*	*D*···*A*	*D*—H···*A*
O8—H8A···O13^ix^	0.84	2.02	2.8584 (19)	172
O8—H8B···O4^iii^	0.85	1.98	2.8028 (18)	160
O9—H9A···O7^iv^	0.86	2.16	2.9932 (16)	164
O9—H9B···O2	0.84	1.83	2.6222 (16)	158
O10—H10A···O7^iii^	0.83	2.04	2.8602 (17)	168
O10—H10B···O3^viii^	0.85	1.84	2.6676 (18)	165
O11—H11A···O7^iv^	0.89	1.89	2.7621 (17)	167
O11—H11B···O3^viii^	0.87	1.92	2.7904 (19)	175
O12—H12A···O1^i^	0.84	2.12	2.9531 (17)	174
O12—H12B···O4^x^	0.89	2.05	2.9076 (18)	162
O13—H13A···O4^iii^	0.84	1.93	2.7277 (18)	157
O13—H13B···O6^ii^	0.88	2.22	2.9597 (17)	142
C7—H7···O11^v^	0.93	2.49	3.371 (2)	157

Symmetry codes: (ix) −*x*+2, −*y*+1, −*z*+1; (iii) −*x*+1, −*y*+1, −*z*; (iv) −*x*+1/2, *y*−1/2, −*z*+1/2; (viii) *x*+1/2, −*y*+1/2, *z*+1/2; (i) −*x*+1, −*y*+1, −*z*+1; (x) *x*+1, *y*, *z*+1; (ii) −*x*+3/2, *y*−1/2, −*z*+1/2; (v) *x*−1/2, −*y*+1/2, *z*−1/2.
